# Assessing the Mandatory Bovine Abortion Notification System in France Using Unilist Capture-Recapture Approach

**DOI:** 10.1371/journal.pone.0063246

**Published:** 2013-05-14

**Authors:** Anne Bronner, Viviane Hénaux, Timothée Vergne, Jean-Luc Vinard, Eric Morignat, Pascal Hendrikx, Didier Calavas, Emilie Gay

**Affiliations:** 1 Agence nationale de sécurité sanitaire de l’alimentation, de l’environnement et du travail (Anses), Unité Epidémiologie du Laboratoire de Lyon, Lyon, France; 2 Centre de coopération internationale en recherche agronomique pour le développement (Cirad), Département ES, UR22, TA C22/E, Montpellier, France; 3 Agence nationale de sécurité sanitaire de l’alimentation, de l’environnement et du travail (Anses), Laboratoire de Santé animale de Maisons-Alfort, Maisons-Alfort, France; 4 Agence nationale de sécurité sanitaire de l’alimentation, de l’environnement et du travail (Anses), Unité de surveillance épidémiologique (Survepi), Direction scientifique des laboratoires, Maisons-Alfort, France; Iowa State University, United States of America

## Abstract

The mandatory bovine abortion notification system in France aims to detect as soon as possible any resurgence of bovine brucellosis. However, under-reporting seems to be a major limitation of this system. We used a unilist capture-recapture approach to assess the sensitivity, i.e. the proportion of farmers who reported at least one abortion among those who detected such events, and representativeness of the system during 2006–2011. We implemented a zero-inflated Poisson model to estimate the proportion of farmers who detected at least one abortion, and among them, the proportion of farmers not reporting. We also applied a hurdle model to evaluate the effect of factors influencing the notification process. We found that the overall surveillance sensitivity was about 34%, and was higher in beef than dairy cattle farms. The observed increase in the proportion of notifying farmers from 2007 to 2009 resulted from an increase in the surveillance sensitivity in 2007/2008 and an increase in the proportion of farmers who detected at least one abortion in 2008/2009. These patterns suggest a raise in farmers’ awareness in 2007/2008 when the Bluetongue Virus (BTV) was detected in France, followed by an increase in the number of abortions in 2008/2009 as BTV spread across the country. Our study indicated a lack of sensitivity of the mandatory bovine abortion notification system, raising concerns about the ability to detect brucellosis outbreaks early. With the increasing need to survey the zoonotic Rift Valley Fever and Q fever diseases that may also cause bovine abortions, our approach is of primary interest for animal health stakeholders to develop information programs to increase abortion notifications. Our framework combining hurdle and ZIP models may also be applied to estimate the completeness of other clinical surveillance systems.

## Introduction

In the context of increasing cross-border movements of people and growing international trade of animals and animal products, efficient and reliable surveillance systems are the basis to assess the actual disease situation in a country or region. Mandatory notification systems are essential in human and animal health as they allow health authorities to react promptly and control the spread of diseases. In France, clinical (or “passive”) surveillance covers 65 animal diseases that may cause important public health or economical impacts; among them, 60% are sporadic or currently exotic. Passive surveillance systems play an important role in early warning systems but their sensitivity in detecting cases has not been routinely evaluated [1].

The mandatory bovine abortion notification system, implemented in France in 1965, aims to detect as soon as possible any resurgence of bovine brucellosis [2]. This highly infectious zoonosis due to *Brucella abortus* (and less frequently to *B. melitensis* and *B. suis*) in cattle affects primarily the reproductive organs of infected animals. In particular, once introduced in a cattle herd, bovine abortion is the main clinical sign of the disease. Bovine brucellosis was endemic to French cattle population in the 1960’s but was then progressively eradicated by vaccination and test and cull management. France was declared as officially free from bovine brucellosis in 2005 [3]. Nevertheless, the recent outbreaks in Belgium and France (April 2012, [4]) reminded that the risk of introduction of bovine brucellosis still persists and emphasized the importance of an effective clinical surveillance system. In addition, the mandatory bovine abortion notification system might be useful for surveillance of other diseases causing bovine abortion. Thus, high abortion rate in ruminants may also be a signal of the introduction of Rift Valley Fever, an acute, zoonotic viral disease that currently circulates in numerous African countries [5]. Similarly, a clinical surveillance of Q fever has been recently implemented in ten departments (administrative units) in France to estimate the prevalence of this zoonosis in cattle and small ruminants herds [6].

In case of bovine abortion, European regulations require farmers to consult their veterinarian who report the abortion to veterinary services and sample the aborted cow for a serological analysis for *Brucella* [7], [8]. Abortion is defined by national regulation as “the expulsion of the fetus or the calf, stillborn or dying less than 48 hours after being born” [7]. About 8 million reproductive cows distributed in 166,000 herds compose the French bovine stock [9]. For the last 5 years, 25 to 30,000 farmers participated in the national mandatory abortion notification system and 50 to 60,000 abortions were reported each year, which correspond to about 20% of the herds and 0.70 to 0.80% of the pregnant cows (considering a barrenness of about 5–10% [10]). However, a recent study of the delay between artificial insemination and calving in dairy cattle estimated that the rates of abortion occurring in mid-pregnancy and late pregnancy were about 6.4% and 5.1%, respectively [11]. “Usual” annual abortion risks have been cited elsewhere to vary from 3 to 5% once cows are beyond 42 days of pregnancy [12]. Although abortions are not always detected (it has been estimated that only 20 to 30% of abortions are detected visually [13]), the low proportion of cases reported to the system suggests that some abortions remain unreported.

Under-reporting of abortions in cattle may impede the early detection of incursions of bovine brucellosis or other infectious agents that may cause abortion. To our knowledge, no study has evaluated the completeness of the register. Estimating the proportion of under-reporting farmers, i.e. who detected at least one abortion but did not report any, and determining the factors that drive the decisions of farmers to report abortions are central goals for public health authorities to assess the performances and sensitivity of the surveillance system.

As often advocated in public health surveillance, data collected by a surveillance system may be analyzed by capture-recapture methods to take into account the fact that some events of interest are not observed. Unilist capture-recapture approach is implemented when data are collected by a single source of observations with repeated entries [14]–[16]. In our case, only herds with reported abortion(s) were “observed” by the mandatory bovine abortion notification system [17], [18]. These data were incomplete because some farmers may have detected abortion(s) but did not report any. Multi-response or mixture models, including zero-inflated Poisson (ZIP) and hurdle models, provide flexible approaches to model the heterogeneity among farmers in the notification process [19].

Our objectives were to assess the sensitivity (i.e. the proportion of farmers who reported at least one abortion among those who detected such events) and representativeness of the mandatory bovine abortion notification system in France during 2006–2011, and to study the factors influencing the notification process. We applied a ZIP model to estimate the proportion of farmers who detected at least one abortion and among them, the proportion of farmers not reporting. We then quantified the effect of the factors influencing the farmers’ probability to report at least one abortion and the number of abortions reported by notifying farmers using a hurdle model.

## Materials and Methods

### Data Sources and Study Population

We extracted data related to abortion notification data from the French national animal health information database SIGAL, including the herd identification number, dates of veterinarian visits (or dates of abortion), and pregnancy stages of the aborted cows. For all farmers, we extracted information about cattle farm location (department), animals (identification number, birth date, sex and breed), and animal movements (herd identification number, date, reason for entry [birth], reason for exit [death]) from the French National Cattle Register.

We considered five reproductive seasons between 2006 and 2011: reproductive season X started on August 1^st^ of year X and ended on July 31^st^ of year X+1 to follow the seasonality of calving, peaking in September/October for dairy cows and in March for beef cows. The study focused on all departments that reported at least one abortion per reproductive season since 2005. A department is a French administrative unit with a mean area of 5,800 km^2^. For each department, we considered bovine farmers who owned at least one reproductive cow per reproductive season. A reproductive cow was defined as a female aged two years and over at the start of a reproductive season; thus, we included 98% of the calving records for each reproductive season. For each reproductive season, a cattle herd was characterized by its department, size, production type and the reported number of abortion(s). Herd size was calculated as the mean number of reproductive cows held per day. Three production types were defined according to the breeds of the animals that composed the herd: beef, dairy, and mixed (combining beef and dairy) cattle herd.

### Multi-response Models

The data consisted on counts of abortions 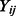 reported by each farmer *i* = 1,2,…, N within a given reproductive season *j*. 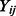 was a random variable, which could take values (0,1,2,…) for each herd. Herds were “observed” (or “captured”) by the mandatory bovine abortion notification system only when farmers reported abortion(s) (i.e. 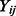
≥1). Thus, the observed proportion of notifying farmers (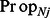) was defined as the ratio of the observed number of farmers who reported at least one abortion to the total number of farmers. However, farmers with no observation (referred as “non-notifying farmers” hereafter) included farmers who detected no abortion whether an abortion occurred or not and farmers who detected abortion(s) but did not report any. Even if abortion notification implied abortion occurrence, detection, and notification itself, we focused only on the detection and notification processes.

Preliminary analyses considering 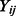 as a simple random Poisson variable did not provide a good fit to the data because it did not account for the excess of zeros [19], [20], due to the presence of farmers who detected no abortion. Therefore, we used multi-response models to account for zero-inflation and to evaluate separately the various processes generating the observations [19].

#### ZIP model

The ZIP model described 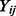 as the result of two random variables: the binary (1/0) realization of the farmers’ probability to detect at least one abortion (unobserved process) 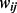 and the number of abortion(s) reported by farmers who detected at least one abortion 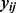 such that 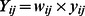 [21] (Figure S1). The probability for a farmer to detect at least one abortion 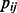 was expressed by a logistic regression (link logit): 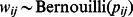. The number of abortion(s) reported by farmers who detected at least one abortion was modeled by a Poisson regression (link log): 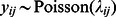, with mean 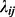.

For farms where no abortion was detected (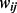
_ = _0), the observed number of reported abortions was zero (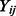 = 0). On the other hand, for farms where at least one abortion was detected (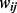
_ = _1), the number of reported abortion(s) was 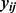 = 0 for farmers not reporting or 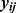 >0 for notifying farmers. Thus, among non-notifying farmers (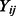 = 0), the ZIP model differentiated farmers who did not detect any abortion (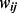
_ = _0) from under-reporting farmers, who detected (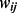
_ = _1) but did not report any abortion. Therefore, the probability for a farmer to report 0 abortion was calculated as 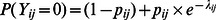. The probability for a farmer to report *k* abortions (*k* = 1,2,…) was 
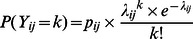
.

For each reproductive season *j*, the proportion of farmers who detected abortion(s) (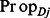) was calculated as the ratio of the number of farmers who detected at least one abortion to the total number of farmers *N*: 
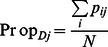
. The proportion of under-reporting farmers (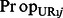) was calculated as the ratio of the number of farmers who detected but did not report abortion(s) to the total number of farmers who detected at least one abortion, 
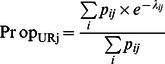
.

#### Hurdle model

The hurdle model described 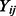 as the result of two random variables: the binary (1/0) realization of the farmers’ probability to report at least one abortion 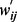 and the number of abortion(s) reported by notifying farmers 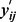 such that 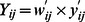 [21] (Figure S1). The probability for a farmer to report at least one abortion 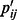 was expressed by a logistic regression (link logit): 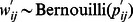. The number of abortion(s) reported by notifying farmers, i.e. farmers who had decided to “cross the hurdle” of the notification process, was modeled by a zero-truncated Poisson regression (link log): 
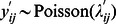
, with mean 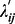.

Once the farmer had decided to report at least one abortion (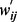 = 1), the number of reported abortion(s) 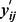 was positive (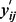 >0). Thus, this model analyzed separately the “zero count” (i.e. non-notifying farmers, 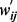 = 0) and counts of one or more reported abortions (i.e. notifying farmers, 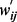 = 1) [19], [22]. With the hurdle model, the probability for a farmer to report 0 abortion was: 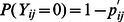, and the probability for a farmer to report *k* abortions (*k* = 1,2,…) was: 
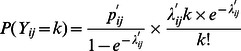
.

### Inference on the Number of Bovine Abortion Notification(s) by Farmers

#### Covariates

For each model, the Bernouilli and (zero-truncated) Poisson responses could be predicted by a set of explanatory variables [23], [24]. We considered the production type of herd (dairy, beef, mixed), herd size (with categories based on quartiles) and reproductive season as categorical variables in the logistic and (zero-truncated) Poisson regressions. In the (zero-truncated) Poisson regression, the herd size was also included as an offset in order to take into account the suspected linear relationship between the number of abortion notifications and herd size (mechanistic effect). The location of the herd (department) and the farmer were studied as random effects. The R MCMCglmm package used to run Bayesian simulations [25] included automatically an additive over-dispersion parameter e (or “residuals”) in both linear predictors as a random effect, for which a residual variance was estimated [26], [27].

We ran the ZIP model for each reproductive season *j* separately in order to estimate for season-specific 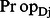 and 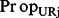. The ZIP models included covariates in the logistic and Poisson regressions, respectively, as
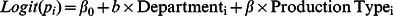



and
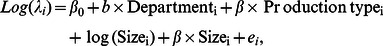
where 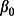 was the intercept, 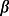 were the covariate coefficients, *b* was the random effect, and *e* corresponded to the residuals.

We ran a single hurdle model including reproductive season as a fixed effect in order to study a potential between-seasons variation in the probability for a farmer to report at least one abortion and in the number of abortion(s) reported by notifying farmers. The hurdle model included covariates in the logistic and truncated-Poisson regressions, respectively, as
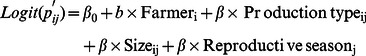



and
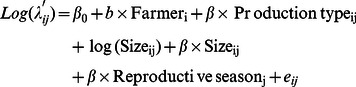



For the logistic regression, we calculated the odds ratio (OR) of each covariate as OR = exp(β). Similarly, for the (zero-truncated) Poisson regressions, we calculated the relative risk (RR) for each covariate, as RR = exp(β) [28].

#### Random sampling

Due to the high number of herds included in the study population (n = 94,640), models were implemented using a stratified randomly selected sample of herds continuously monitored from 2006 to 2011 with department as strata. We selected 10% (n = 9,462) of the entire population of herds for the ZIP model and 5% (n = 4,732) for the hurdle model, to maintain precision while reducing computing time. We checked the robustness of the results by performing a second round of estimations for the selected models with another sample.

#### Priors distribution

We used proper uninformative distributions for hyperparameters, except for the offset (Table 1) [27], [29]. As proposed by Hadfield [24], the variance of the over-dispersion parameter integrated in the logistic part of the ZIP and hurdle models was set to 1 and the fixed effects and variances were rescaled. To investigate the sensitivity of the posterior inference to the prior information, we fitted the selected model using different prior distributions. We assessed the relative changes in the posterior distribution of the parameters by multiplying by 2 the scale of the half-Cauchy prior distributions and by 10 the parameter of the inverse-gamma prior distribution.

**Table 1:** Prior distributions of the hyperparameters for the ZIP and hurdle models.

Hyperparameter	Submodel	Prior distribution
Fixed effects (except the offset)	Logistic regression	*N*(0, 10^10^)
	(truncated-)Poisson regression	*N*(0, 10^10^)
Offset	(truncated-)Poisson regression	*N*(1, 10^−6^)
Standard deviation of the “Department” and Herd effects	Logistic regression	*Half-Cauchy*(0, 5)
	(truncated-)Poisson regression	*Half-Cauchy*(0, 5)
Variance “Over-dispersion parameter”	Logistic regression	Fixed to 1
	(truncated-)Poisson regression	*Invγ*(0.001, 0.001)

*N* refers to a normal distribution and 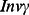 to an inverse gamma distribution. Poisson regression refers to the ZIP model and truncated-Poisson regression to the hurdle model.

#### Models implementation

A total of 800,000 samples were run including a burn-in period of 500,000 and we used a thinning sample of 100. Two simulation chains were obtained and convergence was assessed using the Gelman-Rubin convergence criterion [30]. Models were selected considering the statistical significance of covariates and their convergence. We checked model fit by studying the discrepancy between the observed and predicted numbers of farmers who reported *k* abortion(s) and assessing the posterior mean of the standardized residuals [31].

Posterior distributions of 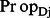 and 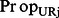 were obtained from each ZIP model. Differences in posterior means among reproductive seasons and types of production were evaluated with a z-test using the significance level α = 0.05. We approximated standard errors by dividing the 95% credible intervals by 3.92 (2×*Z*_0.05_). P-values were adjusted using the Bonferoni method [32].

## Results

### Population Characteristics

**Figure 1:**
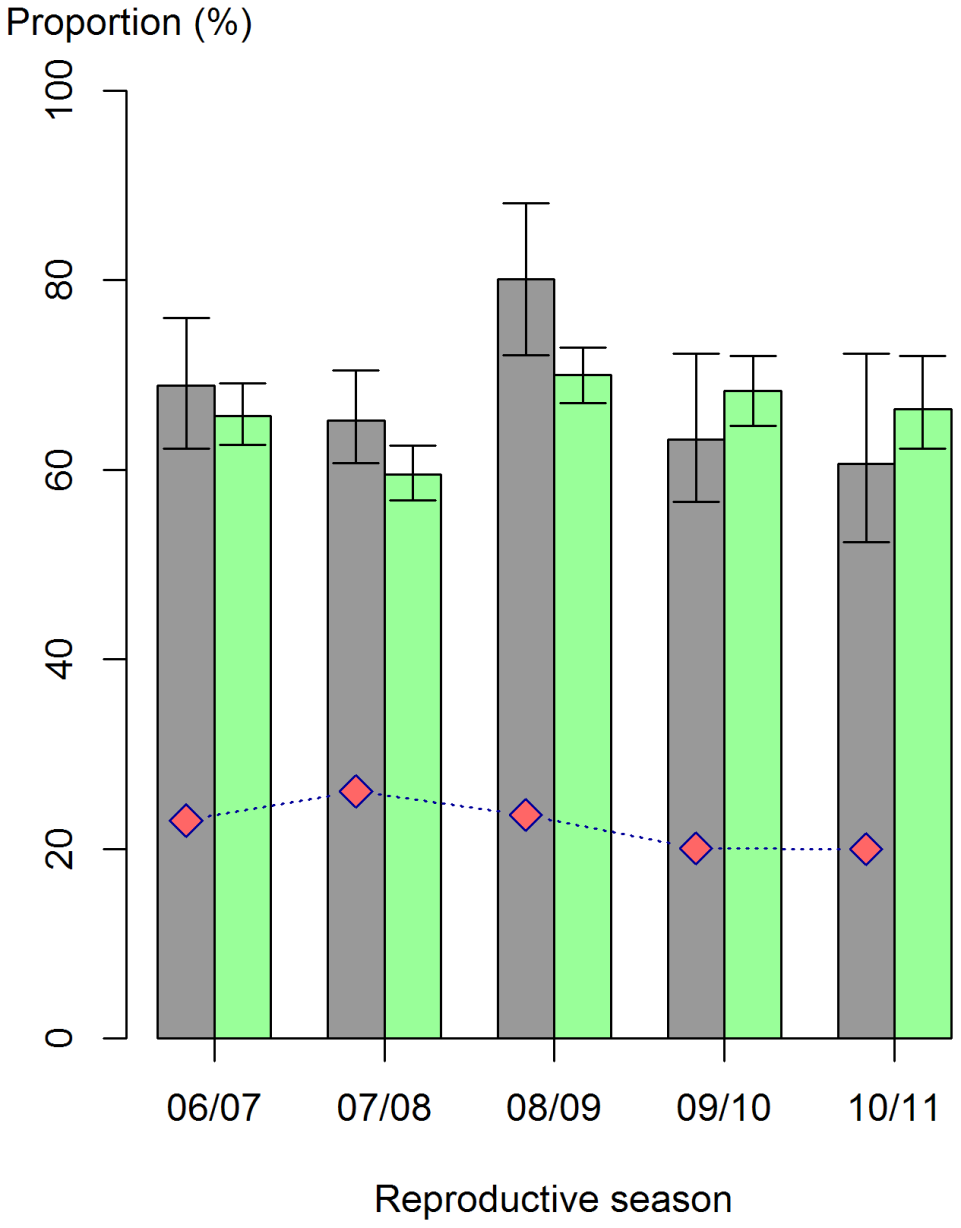
Proportion of farmers who detected, reported or failed to report abortions during the 2006–2011 period. The proportion of notifying farmers (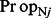; dotted blue line and red diamond shape) was calculated as the ratio of the observed number of farmers who reported at least one abortion to the total number of farmers. The proportion of farmers who detected at least one abortion (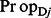; grey) and the proportion of under-reporting farmers (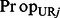; light green) were estimated from the ZIP model. In 2010/2011, 60.6% [95% confidence interval: 52.4–72.2] of farmers detected at least one abortion (i.e. 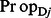;); among them, 20.0% reported at least one abortion (i.e. 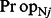). The proportion of under-reporting farmers 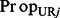
was estimated to be 66.4% [62.2–72.0].

The study population included 94,640 herds from 37 departments (which represented 78.7% of the 120,213 herds registered during the 2006–2011 period), among which 36.5 to 40.0% were beef cattle, 21.1 to 25.3% dairy cattle, and 38.2 to 39.1% mixed cattle depending on the reproductive season. During the study period, 25% of the herds held less than 18 reproductive cows on average, 50% less than 38, and 75% less than 60. On average, there were 37±35 (sd), 39±24, and 52±38 reproductive cows in beef, dairy and mixed cattle, respectively. Between 20.0% (in the 2010/2011 reproductive season) and 26.1% (in 2007/2008) of farmers reported at least one abortion per reproductive season (Figure 1). Among notifying farmers, an average of 60.5% (min-max: 58.3–62.0%) reported one abortion and 21.9% (min-max: 21.3–22.7%) two abortions depending on the reproductive season. 76.5% of reported abortions occurred after 6 months of pregnancy (among the 92.4% of reported abortions for which the information was available). Characteristics of the herds included in the two subsamples used to run the ZIP and hurdle models did not differ from the study population characteristics (Table S1).

### Inferences from the ZIP Model

We found a good fit between the observed number of farmers who reported *k* abortion(s) and the ZIP-model predictions for all reproductive seasons (Figure S2). Model predictions were robust to a change in prior distributions and when analyzing another subsample (results not shown).


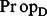
 varied from 60.6% to 80.1% depending on the reproductive season and 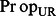 from 59.5% to 70.0% (Figure 1). For all reproductive seasons, 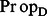 in beef cattle (about 58%) was lower than in dairy cattle (about 84%) but similar to mixed cattle (Tables 2 and 3). In contrast, 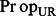 was lower for dairy (about 59%) than beef cattle farmers (about 79%; Table 3). Overall, 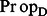 was significantly higher during the 2008/2009 reproductive season compared to other seasons and 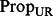
was significantly lower in 2007/2008 than during other reproductive seasons (Figure 1). The number of abortions reported by farmers who detected at least one abortion was influenced by production type and herd size. Among farmers who detected at least one abortion, farmers with dairy cattle reported on average twice more abortions that those with beef cattle (Table 4). When adjusting for the proportional increase in the number of abortions with herd size (using an offset in the model), farmers with 18 to 60 reproductive cows tended to report more abortions than those with less than 18 reproductive cows; in contrast, farmers with more than 60 reproductive cows reported as many abortions as those with less than 18 reproductive cows. The residual variance of the over-dispersion parameter was higher than the variance of the department (Table 4).

**Table 2:** Odds ratios (OR) for the probability of detecting at least one abortion in cattle.

Reproductive season	Production type (OR)		Variance “Department”
	Dairy	Mixed	
2006/2007	**4.9 [1.52–30.89]**	1.3 [0.78–2.21]	0.15 [0–0.39]
2007/2008	**2.49 [1.49–4.04]**	1.32 [0.9–1.9]	0.31 [0.07–0.64]
2008/2009	**8.88 [1.5–146.06]**	1.17 [0.54–3.02]	0.95 [0.05–2.29]
2009/2010	**3.53 [1.57–8.09]**	1.44 [0.69–2.52]	0.2 [0–0.51]
2010/2011	**5.75 [2.46–32.4]**	**1.84 [1.09–2.85]**	0.35 [0.02–0.81 ]

The probability for a farmer to detect at least one abortion (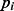) was modeled by the logistic regression of the ZIP model. 95% credible intervals are mentioned in square brackets and bolded values indicate significant differences (i.e. credible interval not including 1.00). Beef cattle herds were considered as the reference for each reproductive season. Variance corresponded to the amount of variation among departments.

**Table 3:** Proportion of farmers who detected and failed to report abortion by production type.

Reproductive season	Proportion	Beef	Dairy	Mixed
2006/2007	detection	59.3 [49.6–69.5]	86.6 [78.0–99.5]	66.3 [59.3–74.1]
	under-reporting	78.1 [74.9–81.5]	59.9 [55.8–64.4]	60.0 [56.1–63.9]
2007/2008	detection	57.2 [49.7–64.9]	78.2 [71.3–85.1]	65.0 [59.6–69.9]
	under-reporting	73.4 [70.5–76.6]	52.3 [48.8–55.9]	53.1 [50–56.4]
2008/2009	detection	74.0 [63.2–87.5]	93.8 [85.5–99.9]	78.1 [69.2–88.4]
	under-reporting	82.6 [79.8–85.2]	62.1 [58.8–65.2]	63.7 [59.7–67.6]
2009/2010	detection	53.5 [42.4–68.4]	80.3 [71.2–90]	63.1 [55.4–70.8]
	under-reporting	81.3 [77.4–85.2]	61.2 [57.4–65.3]	62.2 [58–66.3]
2010/2011	detection	46.8 [36.1–61.4]	82.1 [72.4–99.6]	63.1 [55.5–72.2]
	under-reporting	80.0 [75.7–84.9]	60.8 [56.4–67.4]	59.9 [55.4–64.9]

The proportion of farmers who detected at least one abortion (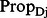 *)* was the ratio of the number of farmers who detected at least one abortion to the total number of farmers. The proportion of under-reporting farmers (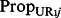) was the ratio of the number of farmers who detected but did not report abortion to the number of farmers who detected at least one abortion. Both proportions were estimated from the ZIP model. 95% credible intervals are mentioned in square brackets.

**Table 4:** Relative risks (RR) for the number of abortion notification(s) by farmers who detected abortion(s).

Variables		2006/2007	2007/2008	2008/2009	2009/2010	2010/2011
Production type (RR)	Dairy	**1.83 [1.43–2.32]**	**1.85 [1.53–2.22]**	**2.17 [1.79–2.67]**	**2.08 [1.60–2.84]**	**2.04 [1.61–2.68]**
	Mixed	**1.51 [1.20–1.87]**	**1.5 [1.27–1.77]**	**1.69 [1.38–2.07]**	**1.59 [1.19–2.14]**	**1.58 [1.24–2.09]**
Herd size (RR)	[18–38[	1.18 [0.97–1.46]	**1.24 [1.03–1.52]**	**1.57 [1.25–1.95]**	**1.36 [1.06––1.74]**	**1.34 [1.05–1.70]**
	[38–60[	1.09 [0.90–1.35]	**1.25 [1.04–1.5]**	**1.44 [1.15–1.78]**	**1.38 [1.10–1.76]**	1.24 [0.98–1.57]
	> = 60	0.94 [0.76–1.15]	1.02 [0.84–1.23]	1.23 [0.99–1.54]	1.12 [0.89–1.43]	1.05 [0.83–1.31]
Variance “Department”		0.11 [0.04–0.19]	0.07 [0.03–0.13]	0.17 [0.06–0.31]	0.1 [0.03–0.17]	0.11 [0.04–0.20]
Variance “Overdispersion parameter”		0.62 [0.48–0.78]	0.49 [0.38–0.59]	0.71 [0.53–0.85]	0.66 [0.50–0.83]	0.7 [0.50–0.95]

The number of abortion notification(s) by farmers who detected at least one abortion (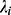) was modeled by the Poisson regression of the ZIP model. 95% credible intervals are mentioned in square brackets and bolded values indicate significant differences (i.e. credible interval not including 1.00). Beef cattle farmers with less than 18 reproductive cows during the 2007/2008 reproductive season were considered as the reference. Variance corresponded to the amount of variation associated with the corresponding random variable.

### Inferences from the Hurdle Model

The hurdle model provided a good fit to the observed number of farmers who reported k abortion(s) (Figure S2). Model predictions were robust to a change in prior distributions and when analyzing another subsample (results not shown).

The probability for a farmer to report at least one abortion was higher for dairy than for beef cattle herds (OR = 3.94 [3.40–4.54]), and increased with the number of reproductive cows (Table 5); it was higher in the 2007/2008 reproductive season than in other seasons (Table 5). The number of abortion notifications was 1.77 [1.55–2.00] higher for notifying farmers with dairy than beef cattle herds (Table 6). When adjusting for the proportional increase in the number of abortions with herd size (using an offset in the model), the ability to report abortions was equal among farmers whatever the size of their herds (Table 6). The number of abortion notifications by notifying farmers was higher in 2007/2008 than in other reproductive seasons (Table 6).

**Table 5:** Odds ratios (OR) for the probability of reporting abortion(s) in cattle.

Variable		Estimation
Production type (OR)	Dairy	**3.94 [3.40–4.54]**
	Mixed	**2.27 [2.01–2.59]**
Size (OR)	[18–38[	**6.49 [5.35–7.79]**
	[38–60[	**12.16 [10.02–14.70]**
	≥60	**16.13 [13.33–19.70]**
Reproductive season (OR)	2006/2007	**0.79 [0.71–0.88]**
	2008/2009	**0.81 [0.72–0.90]**
	2009/2010	**0.61 [0.55–0.69]**
	2010/2011	**0.61 [0.54–0.68]**
Variance “Farmers”		1.98 [2.19–1.77]

The probability for a farmer to report at least one abortion (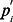) was modeled by the logistic regression of the hurdle model. 95% credible intervals are mentioned in square brackets and bolded values indicate significant differences (i.e. credible interval not including 1.00). Beef cattle farmers with less than 18 reproductive cows during the 2007/2008 reproductive season were considered as the reference. Variance corresponds to the amount of variation among farmers.

**Table 6:** Relative risks (RR) for the number of abortion notification(s) by notifying farmers.

Variable		Estimation
Production type (RR)	Dairy	**1.77 [1.55–2.00]**
	Mixed	**1.45 [1.28–1.63]**
Size (RR)	[18–38[	1.06 [0.73–1.51]
	[38–60[	0.93 [0.64–1.34]
	≥60	0.72 [0.49–1.02]
Reproductive season (RR)	2006/2007	**0.85 [0.76–0.94]**
	2008/2009	**0.87 [0.79–0.97]**
	2009/2010	**0.83 [0.74–0.92]**
	2010/2011	**0.79 [0.71–0.89]**
Variance “Farmers”		0.27 [0.21–0.33]
Variance “Overdispersion parameter”		0.26 [0.19–0.32]

The number of abortion notification(s) by farmers who reported at least one abortion (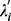) was modeled by the zero-truncated Poisson regression of the hurdle model. 95% credible intervals are mentioned in square brackets and bolded values indicate significant differences (i.e. credible interval not including 1.00). Beef cattle farmers with less than 18 reproductive cows during the 2007/2008 reproductive season were considered as the reference. Variance corresponded to the amount of variation associated with the corresponding random variable.

## Discussion

Given that most notifications concerned late abortions, our results are representative of abortions that occurred during the last 4 months of pregnancy. From 2006 to 2011, about 23% of farmers reported at least one abortion, although our models predicted that 68% detected at least one abortion. Thus, 45% of the farmers detected at least one abortion but did not report any, which means that among farmers who detected at least one abortion, 66% did not report any. The proportion of farmers who reported at least one abortion among those who detected such events was more important for dairy than beef cattle. Once a farmer had detected or reported at least one abortion, the number of abortion notification(s) was higher for dairy than beef cattle farmers.

### Unilist Capture-recapture Assumptions

Unilist capture-recapture approach assumes capture homogeneity and independence between capture and recapture [18]. In our study, validating the homogeneity assumption required that the number of detected abortions and the probability of reporting them were the same between herds presenting the same set of covariates. As bovine abortions are generally sporadic events, we expected only few variations in the number of detected abortions between herds. But because the probability of detecting abortion(s) in a farm certainly depends on the frequency of contacts between farmers and their herds, we included the production type as a covariate in both models. In addition, we considered potential variation in the probability of reporting abortion(s) among farmers by including an over-dispersion parameter in the ZIP model and a random effect “farmer” in the hurdle model. Secondly, in the capture-recapture approach the reporting probabilities of successive abortions within a cattle herd are assumed to be independent. This assumption was admissible as most of abortions are sporadic events. However, the dependence between captures in herds exposed to epizootic abortions, such as observed during neosporosis outbreaks, may cause an underestimation of the proportion of under-reporting farmers [18], [33].

### Factors Driving Farmers’ Decision to Report Abortions

Our analysis predicted that, during each reproductive season and within the 37 departments included in the study, 19.9 to 39.4% of farmers did not detect any abortion in their herd and among farmers who did detect abortions, 59.5 to 70.0% did not report any. This high proportion of under-reporting farmers is surprising as the veterinarian visit in case of abortion is financed by veterinary services in France. We suspect that the absence of known brucellosis outbreak during 2006–2011 did not encourage farmers to report clinical suspected cases. In addition, farmers may not get worried about the economical impact of abortions as long as these are sporadic: farmers may contact their vet for a diagnosis only if the number of abortions goes over a “threshold” that may vary from one farmer to another. However, our results indicate some variations among departments in the probability of abortion notification likely because the mandatory bovine abortion notification system is implemented at the department level and some local animal health associations encourage farmers to diagnose other diseases (in addition to brucellosis) that may have an economic impact.

Our analysis indicated that the probability for a farmer to report at least one abortion was lower for beef that for dairy cattle herds: the proportion of farmers who detected at least one abortion was lower for beef than dairy cattle herds, and farmers with beef cattle herd were less prone to report detected abortion(s) than those with dairy cattle. Once farmers decided to report at least one abortion, the number of abortion notifications was more important for dairy than for beef cattle farmers. These results are unexpected as calves are the main product in beef cattle herds. However, the lower watchfulness of beef cattle during the grazing period reduce the farmers’ ability to detect abortions (whether fewer abortions occur in these herds or not). Accordingly, beef cattle farmers are not used as much as dairy cattle farmers to health related and technical checks and thus may be less prone to contact their vet.

### Variations among Reproductive Seasons

Our results indicated that the proportion of non-notifying farmers was lower in 2007/2008 compared to other reproductive seasons. Although the predicted proportion of farmers who detected at least one abortion did not change, we found that farmers were more prone to report detected abortions during that reproductive season. However, in 2008/2009, the proportion of non-notifying farmers had increased again, in spite of a higher number of farmers who detected at least one abortion. Several events during these years may explain these findings. First, the implementation in 2005 of an annual official vet visit to inform farmers about the abortion issue may have increased farmers’ awareness of notification in 2007/2008. Besides, following the introduction of Bluetongue Virus (BTV) in 2006, the number of cases started to rise dramatically with 7,607 outbreaks reported in the 2007/2008. The coverage by national medias about the suspected role of BTV in bovine abortions may have also encouraged abortion notification [34], [35]. In 2008/2009, the proportion of farmers who detected at least one abortion increased while the number of BTV outbreaks peaked (12,243 reported outbreaks) [36], suggesting a direct impact of the virus on abortion occurrence [11]. The concurrent increase of the proportion of under-reporting farmers (Figure 1) may be explained by the fact that the role of vaccination against BTV was debated at that time, and tensions between farmers and vets may have discouraged farmers to notify. Afterwards, the decline in BTV cases to 13 cases in 2009/2010 and none in 2010/2011 may explain the lower proportion of farmers who detected at least one abortion those years.

### Advantages and Limits of the Study

Combining hurdle and ZIP models provides a comprehensive framework to examine the sensitivity of the bovine abortion notification system and the factors influencing the farmers’ decisions to report these events. These two models complement each other: by accounting for the latent heterogeneity in detection, the ZIP model provided an unbiased estimate of the proportion of under-reporting farmers; on the other side, the hurdle model allowed to investigate the decision-making process of abortion notification.

We were not able to distinguish the relative influence of the probabilities of abortion occurrence, detection and notification. Knowing the frequency of abortion occurrence in cattle is challenging because the absence of registered calf for a reproductive cow during a given reproductive season may result from a fertility problem, or failure of the artificial insemination (AI) or natural breeding. Further analyses combining information from additional sources such as the dates of AI, mating period or intervals between calving would help evaluating the frequency of abortions in cattle. Moreover, our analysis does not enable to estimate the proportion of herds where abortions occurred: our estimates do not consider farms where abortion(s) occurred but remained undetected. However, it is highly probable that most of the herds face abortion(s). Last, investigating the variations of the proportion for farmers to detect at least one abortion and the proportion of under-reporting farmers among departments would allow evaluating the impact of department-specific actions to increase awareness of veterinarians and farmers at local level. We evaluated the effect of covariates that were available and routinely collected at the national level. However, besides production type and herd size, other factors may play a major role in the notification process. The characteristics of the farmer (age, experience, education) or herd management may influence the ability of the farmer to detect abortions or his willingness to report them. Accordingly, a part of the between-season variability in the number of abortions reported by a farmer remains unexplained, and could be due to a change in his perception and attitudes towards the abortion issue over time. Further studies evaluating the rate of abortions occurrence in each production type and investigating the factors that influence the farmers’ decisions to report or not an abortion would help understanding our finding.

### Assessing the Mandatory Bovine Abortion Notification System

The sensitivity of the mandatory bovine abortion notification system varied from 30.0 to 40.5% depending on the reproductive season [37]. This surveillance system is not fully representative as it does not accurately describe the proportion of farmers detecting bovine abortions over time and its distribution in the population by production type [38]: the proportion of beef cattle farmers is under-represented in the mandatory bovine abortion notification system compared to dairy cattle farmers.

Given the high proportion of under-reporting farmers, will the surveillance system detect outbreaks of brucellosis or other critical diseases? Our results suggest that the early detection of any introduction or resurgence of brucellosis may be difficult. In particular, our findings concern all farms in activity since 2005, and we may expect a higher proportion of under-reporting farmers among those who started or stopped their activity during the study period. Difficulties in keeping actors participating in a notification system have already been observed in public health surveillance systems: under-reporting of notifiable diseases was estimated to reach 90% in some cases [39] and the knowledge, attitudes and beliefs of various actors were the main drivers of the decision to report or not [39], [40]. Nevertheless, in contrast to active surveillance which is restricted to a certain time frame and is resource-consuming, passive surveillance presents the advantages of being ongoing, cost-saving and prompt to detect an outbreak. In 2012, one of the two brucellosis outbreaks reported in France was detected thanks to clinical surveillance. In Great Britain, a simulation study of brucellosis spread in cattle under several testing regimes found that suppressing abortion notifications would have a major effect on the rate of spread of infection before detecting a brucellosis outbreak [41]. Beside early detection, clinical surveillance has been suggested to be one of the main way to identify secondary outbreaks once the disease was introduced because disease awareness of animal health actors increases the report of suspect cases [35].

To our knowledge, this study is the first to quantify the proportion of under-reporting farmers and evaluate the factors that influence the notification process of a specific health-related event. As underlined by the Centre for Disease Control, sensitivity and representativeness of public health surveillance systems are the major attributes of the system to assess when a surveillance system is evaluated [38]. Our approach may be applied to other clinical surveillance systems to help identify the ways to increase actors’ awareness and improve the notification of suspected cases.

## Supporting Information

Figure S1
**Graphical representation of the two processes modeled by the ZIP and hurdle multi-response models.** The ZIP model analyzed notification data (i.e., counts of abortions 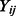 reported by each farmer *i* within a given reproductive season *j*) as the result of two processes: 1) an unobserved probability for farmers to detect at least one abortion (Bernouilli regression) and 2) the number of notifications by farmers who detected at least one abortion (Poisson regression). We estimated the proportion of under-reporting farmers (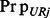) as the ratio of the number of farmers who detected but did not report abortions to the number of farmers who detected at least one abortion. The hurdle model considered 1) the probability for farmers to report at least one abortion (Bernouilli regression) and 2) the number of notifications by notifying farmers (zero-truncated Poisson regression).(DOCX)

Figure S2
**Posterior realization of the discrepancy statistic function** ***Dk*** **under the ZIP and hurdle models.** The predictions 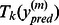 from the ZIP and hurdle models were calculated for *m* = 1,2,….3000 simulated samples. The discrepancy 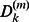 between the observed 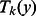 and the predicted number of farmers who reported *k* abortion(s) 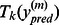 was calculated as 
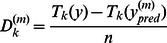
, *n* the number of farms per reproductive season [31]. The model underestimated the number of farmers if 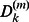
was positive and overestimated it if 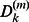
was negative. Figures present the posterior realization of 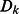 under the ZIP model for the reproductive season 2007/2008 (plots were similar for other reproductive seasons) and under the hurdle model.(DOCX)

Table S1 **Comparisons of the observed probability of reporting abortion(s) and herd characteristics between the samples used for the ZIP and hurdle models analyses (n = 9,462 and n = 4,732 respectively) and the original data set (n = 94,640).** Chi square tests comparing distributions between each subsample and the original data set were not significant.
(XLSX)

## References

[1] Hadorn D, Haracic SS, Stärk K (2008) Comparative assessment of passive surveillance in disease-free and endemic situation: example of *Brucella melitensis* surveillance in Switzerland and in Bosnia and Herzegovina. BMC Veterinary Research 4: 1–9. 19099610 10.1186/1746-6148-4-52 PMC2626581

[2] Fediaevsky A, Dufour B, Garin-Bastuji B (2011) Maintaining vigilance against bovine brucellosis in France in 2010. Bulletin épidémiologique Santé animale- alimentation 46: 10–14.

[3] Anonymous (2005) Commission Decision 2005/764/EC of 28 October 2005 amending Decision 93/52/EEC as regards the declaration that the province of Grosseto in the Region of Toscana in Italy is free of brucellosis (*B. melitensis*) and Decision 2003/467/EC as regards the declaration that France is free of bovine brucellosis. Available: http://eur-lex.europa.eu/fr/index.htm. Accessed 2013 Apr 9.

[4] OIE website. Country reports. Available: http://www.oie.int/wahis_2/public/wahid.php/Countryinformation/Countryreports. Accessed 2013 Apr 9.

[5] Efsa: Opinion of the Scientific Panel on Animal Health and Welfare on a request from the Commission related to “The risk of rift valley fever incursion and its persistence within the Community”. 130 2005: 130.

[6] Anonymous (2012) Arrêté du 13 août 2012 relatif à la constitution d’un dispositif pilote de surveillance de la fièvre Q dans des départements en élevages bovins, ovins et caprins. Available: http://www.legifrance.gouv.fr/. Accessed 2013 Apr 9.

[7] Anonymous (1965) Article R. 223-79 du Code rural et de la pêche maritime. Available: http://www.legifrance.gouv.fr/. Accessed 2013 Apr 9.

[8] Anonymous (1964) Council Directive 64/432/EEC of 26 June 1964 on animal health problems affecting intra-community trade in bovine animals and swine. Available: http://eur-lex.europa.eu/fr/index.htm. Accessed 9 April 2013.

[9] Agreste website. Principaux cheptels selon leur effectif en 2007. Available: http://agreste.agriculture.gouv.fr/IMG/pdf/structure2008T13-2.pdf. Accessed 2013 Apr 9.

[10] Johnson MH, Notter DR (1987) Simulation of genetic control of reproduction in beef cows. I. Simulation model. J Anim Sci 65: 68–75. 3610883 10.2527/jas1987.65168x

[11] Nusinovici S, Seegers H, Joly A, Beaudeau F, Fourichon C (2012) Increase in the occurence of abortions associated with exposure to the Bluetongue virus serotype 8 in naïve dairy herds. Theriogenology, in press. 10.1016/j.theriogenology.2012.05.010 22763077

[12] Hovingh E (2009) Abortion in dairy cattle.II.Diagnosing and preventing abortion problems. Available: http://pubs.ext.vt.edu/404/404-289/404-289.html. Accessed 2013 Apr 9.

[13] Forar A, Gay J, Hancok D (1995) The frequency of endemic fetal loss in dairy cattle: a review. Theriogenology 43: 989–1000. 16727686 10.1016/0093-691x(95)00063-e

[14] Vilas VDR, Böhning D (2008) Application of one-list capture-recapture models to scrapie surveillance data in Great Britain. Preventive veterinary medicine 85: 253–266. 18355934 10.1016/j.prevetmed.2008.02.003

[15] Vergne T, Calavas D, Cazeau G, Durand B, Dufour B, et al (2012) A bayesian zero-truncated approach for analysing capture-recapture count data from classical scrapie surveillance in France. Preventive veterinary medicine 105: 127–135. 22421503 10.1016/j.prevetmed.2012.02.014

[16] Chao A, Tsay P, Lin S-H, Shau W-Y, Chao D-Y (2001) The applications of capture-recapture models to epidemiological data. Statistics in medicine 20: 3123–3157. 11590637 10.1002/sim.996

[17] Gallay A, Vaillant V, Bouvet P, Grimont P, Desenclos JC (2000) How many foodborne outbreaks of *Salmonella* infection occurred in France in 1995? Application of the capture-recapture method to three surveillance systems. Am J Epidemiol 152: 171–177. 10909954 10.1093/aje/152.2.171

[18] Hook E, Regal R (1995) Capture-Recapture methods in epidemiology: methods and limitations. Epidemiologic Reviews 17: 243–264. 8654510 10.1093/oxfordjournals.epirev.a036192

[19] Cameron A, Trivedi P (1998) Regression analysis of count data. New York: Cambridge University Press.

[20] Rodrigues-Motta M, Gianola Heringstad, Rosa Chang (2007) A zero-inflated poisson model for genetic analysis of the number of mastitis cases in norwegian red cows. American Dairy Science Association 90: 5306–5315. 10.3168/jds.2006-898 17954771

[21] Zorn CJ (1996) Evaluating zero-inflated and hurdle poisson specifications. Midwest Political Science Association.

[22] Boucher JP, Denuit M, Guillén M (2008) Risk classification for claim counts: a comparative analysis of various zero-inflated mixed poisson and hurdle models. Available: http://www.scor.com/en/sgrc/pac/claims/item/1354.html?lout=sgrc. Accessed 2013 Apr 9.

[23] Lambert D (1992) Zero-inflated Poisson regression, with an application to defects in manufacturing. Technometrics 34: 1–14.

[24] Cameron A, Trivedi (1998) Regression analysis of count data. New York: Cambridge University Press.

[25] R: A language and environment for statistical computing. R Foundation for Statistical Computing, Vienna, Austria, ISBN 3-900051-07-0 Available http://www.R-project.org. Accessed 2013 Apr 9.

[26] Hadfield J (2010) MCMC methods for multi-response generalised linear mixed models:The MCMCglmm R package. Journal of Statistical Software 33: 1–22.20808728

[27] Hadfield J (2013) Package “MCMCglmm”. Available: http://cran.r-project.org/web/packages/MCMCglmm/MCMCglmm.pdf. Accessed 2013 Apr 9.

[28] Bouyer J, Hémon D, Cordier S, Derriennic F, Stücker I, et al. (1995) Epidémiologie: Principes et méthodes quantitatives. Paris.

[29] Gelman A (2006) Prior distributions for variance parameters in hierarchical models. Bayesian Analysis 1: 515–533.

[30] Gelman A, Rubin DB (1992) Inference from iterative simulation using multiple sequences. Statistical Science 7: 457–472.

[31] Rodrigues-Motta Gianola, Heringstad Rosa (2007) Chang (2007) A zero-inflated Poisson model for genetic analysis of the number of mastitis cases in norwegian red cows. American Dairy Science Association 90: 5306–5315. 10.3168/jds.2006-898 17954771

[32] Dagnelie P (1998) Statistique théorique et appliquée. Tome 1. Statistique descriptive et bases de l’inférence statistique. Bruxelles: De Boeck.

[33] Dubey J, Schares G (2011) Neosporosis in animals - The last five years. Veterinary Parasitology, 180. 10.1016/j.vetpar.2011.05.031 21704458

[34] Moutou F (2002) Epidemiological basis useful for the control of foot-and-mouth disease. Comparative immunology, microbiology and infectious diseases 25: 321–330. 10.1016/s0147-9571(02)00029-2 12365808

[35] Elbers A, Stegeman A, Moser H, Ekker M, Smak J, et al (1999) The classical swine fever epidemic 1997–1998 in the Netherlands: descriptive epidemiology. Preventive veterinary medicine 42: 157–184. 10619154 10.1016/s0167-5877(99)00074-4

[36] Pioz M, Guis H, Calavas D, Durand B, Abrial D, et al (2011) Estimating front-wave velocity of infectious diseases: a simple, efficient method applied to bluetongue. Veterinary Research 42: 42–60. 21507221 10.1186/1297-9716-42-60 PMC3090993

[37] Weinstein MC, Fineberg H (1980) Clinical decision analysis. Philadelphia: Saunders.

[38] CDC (2001) Updated guidelines for evaluating public health surveillance systems - Recommendations from the Guidelines Working Group. Available: http://www.cdc.gov/mmwr/preview/mmwrhtml/rr5013a1.htm. Accessed 2013 Apr 9. 18634202

[39] Figueiras A, Lado E, Fernandez S, Hervada X (2004) Influence of physicians’ attitudes on under-notifying infectious diseases: a longitudinal study. Public Health 118: 521–526. 15351226 10.1016/j.puhe.2003.12.015

[40] Elbers A, Gorgievski-Duijvesteijn M, Zarafshani K, Koch G (2010) To report or not to report: a psychosocial investigation aimed at improving early detection of avian influenza outbreaks. Revue scientifique et technique (OIE) 29: 435–449. 10.20506/rst.29.3.1988 21309445

[41] England T, Kelly L, Jonesa R, MacMillanc A, Wooldridgea M (2004) A simulation model of brucellosis spread in british cattle under several testing regimes. Preventive veterinary medecine 63: 63–73. 10.1016/j.prevetmed.2004.01.009 15099717

