# Supplementary figures and images for: Assessing the Mandatory Bovine Abortion Notification System in France Using Unilist Capture-Recapture Approach
Source: PLoS One. 2013 May 14;8(5):e63246. doi: 10.1371/journal.pone.0063246 (PMC3653928; doi:10.1371/journal.pone.0063246)

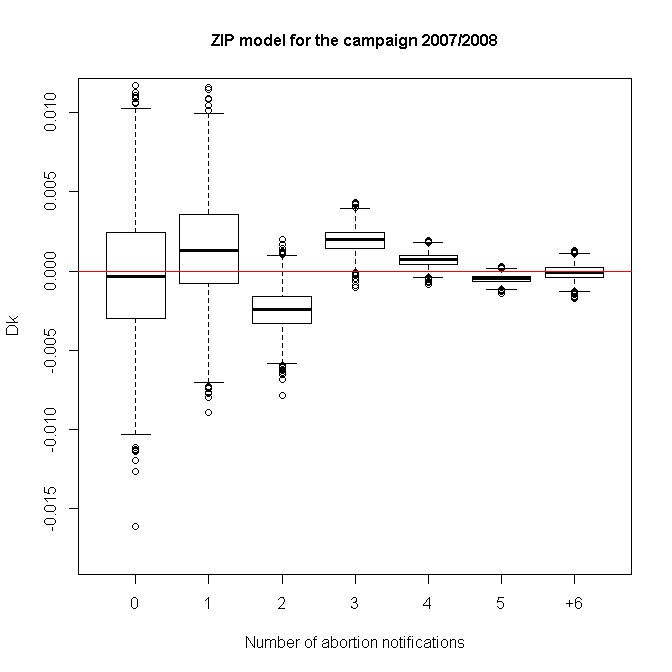

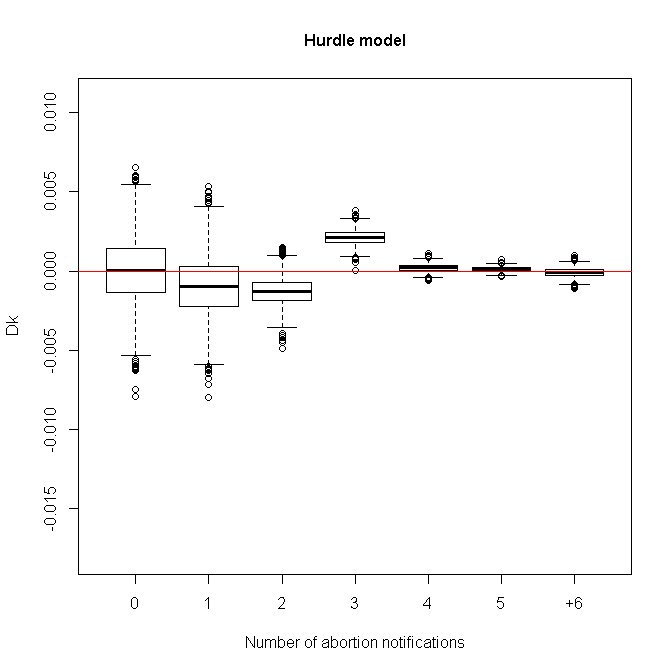

Supplement: Figure S2 — Posterior realization of the discrepancy statistic function Dk under the ZIP and hurdle models. The predictions from the ZIP and hurdle models were calculated for m = 1,2,….3000 simulated samples. The discrepancy between the observed and the predicted number of farmers who reported k abortion(s) was calculated as , n the number of farms per reproductive season [31]. The model underestimated the number of farmers if was positive and overestimated it if was negative. Figures present the posterior realization of under the ZIP model for the reproductive season 2007/2008 (plots were similar for other reproductive seasons) and under the hurdle model. (DOCX) [file pone.0063246.s002.docx]
