# Supplementary material for: Assessing the Mandatory Bovine Abortion Notification System in France Using Unilist Capture-Recapture Approach
Source: PLoS One. 2013 May 14;8(5):e63246. doi: 10.1371/journal.pone.0063246 (PMC3653928; doi:10.1371/journal.pone.0063246)
Supplement: Supplementary file 2 [file pone.0063246.s001.docx]

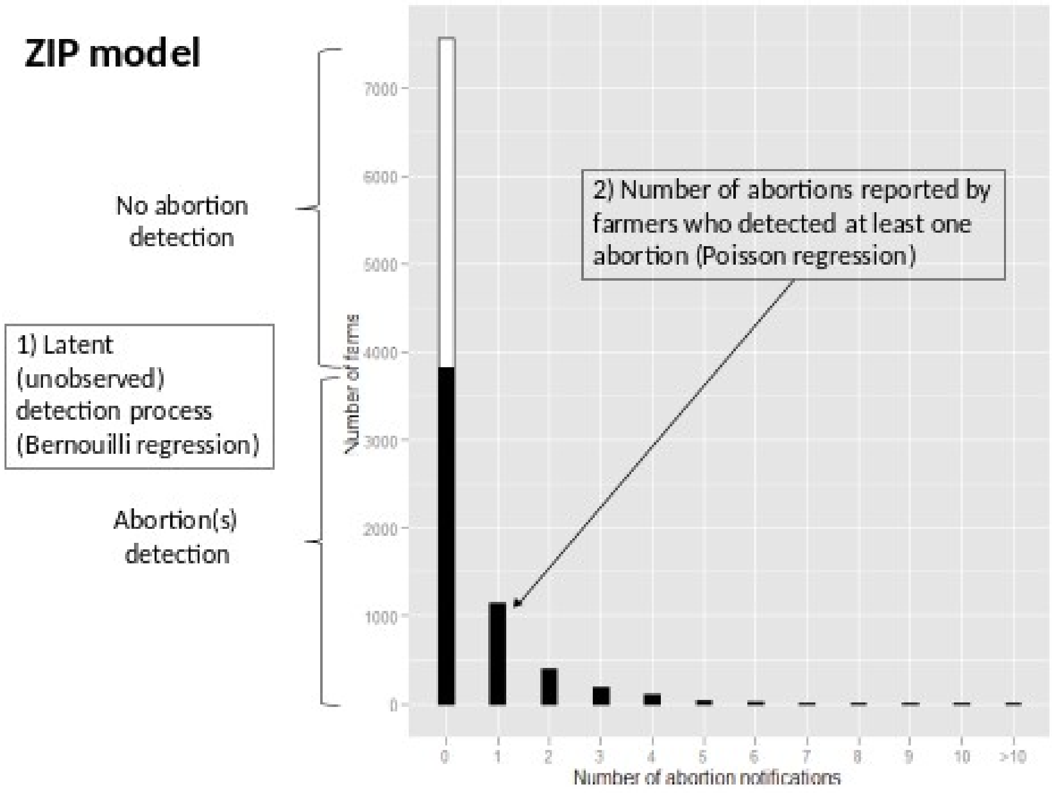
ZIP model
No abortion detection
1) Latent (unobserved) detection process (Bernouilli regression)
Abortion(s) detection
2) Number of abortions reported by farmers who detected at least one abortion (Poisson regression)
Number of farms
Number of abortion notifications

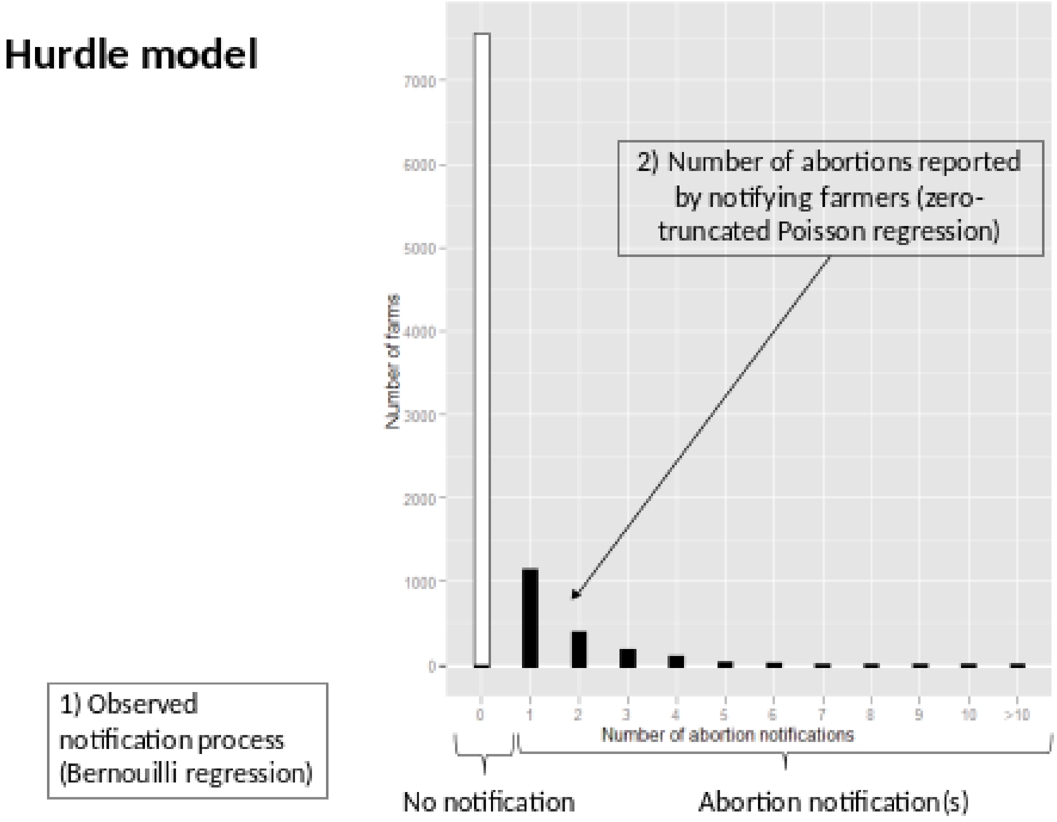
Hurdle model
2) Number of abortions reported by notifying farmers (zero-truncated Poisson regression)
Number of farms
Number of abortion notifications
1) Observed notification process (Bernouilli regression)
No notification
Abortion notification(s)
